# The Spike Mutants Website: A Worldwide Used Resource against SARS-CoV-2

**DOI:** 10.3390/ijms232113082

**Published:** 2022-10-28

**Authors:** Isabella Romeo, Ingrid Guarnetti Prandi, Emanuela Giombini, Cesare Ernesto Maria Gruber, Daniele Pietrucci, Stefano Borocci, Nabil Abid, Anna Fava, Andrea R. Beccari, Giovanni Chillemi, Carmine Talarico

**Affiliations:** 1 Dipartimento di Scienze della Salute, Università Magna Græcia di Catanzaro, Campus “S. Venuta”, Viale Europa, 88100 Catanzaro, Italy; 2 Net4Science Academic Spin-Off, Università Magna Graecia di Catanzaro, Campus “S. Venuta”, Viale Europa, 88100 Catanzaro, Italy; 3 Department for Innovation in Biological, Agro-Food and Forest Systems (DIBAF), University of Tuscia, Via S. Camillo de Lellis s.n.c., 01100 Viterbo, Italy; 4 Laboratory of Virology, INMI Lazzaro Spallanzani IRCCS, Via Portuense 292, 00149 Roma, Italy; 5 Institute of Biomembranes, Bioenergetics and Molecular Biotechnologies (IBIOM), CNR, 70126 Bari, Italy; 6 Laboratory of Transmissible Diseases and Biological Active Substances LR99ES27, Faculty of Pharmacy, University of Monastir, Rue Ibn Sina, Monastir 5000, Tunisia; 7 High Institute of Biotechnology of Monastir, Department of Molecular and Cellular Biology, University of Monastir, Monastir 5000, Tunisia; 8 Dompé Farmaceutici SpA, Via Tommaso De Amicis, 95, 80131 Napoli, Italy

**Keywords:** spike, SARS-CoV-2, COVID-19, variants, molecular dynamics, mutations

## Abstract

A large number of SARS-CoV-2 mutations in a short period of time has driven scientific research related to vaccines, new drugs, and antibodies to combat the new variants of the virus. Herein, we present a web portal containing the structural information, the tridimensional coordinates, and the molecular dynamics trajectories of the SARS-CoV-2 spike protein and its main variants. The Spike Mutants website can serve as a rapid online tool for investigating the impact of novel mutations on virus fitness. Taking into account the high variability of SARS-CoV-2, this application can help the scientific community when prioritizing molecules for experimental assays, thus, accelerating the identification of promising drug candidates for COVID-19 treatment. Below we describe the main features of the platform and illustrate the possible applications for speeding up the drug discovery process and hypothesize new effective strategies to overcome the recurrent mutations in SARS-CoV-2 genome.

## 1. Introduction

As of August 2022, severe acute respiratory syndrome coronavirus 2 (SARS-CoV-2), the causative agent of COVID-19, accounted for more than 599 million infections and more than six million deaths worldwide (https://covid19.who.int, accessed on 1 October 2020). The incessant rise in the number of cases despite the development of vaccines and the resulting immunization process reflects the impact of new variants of SARS-CoV-2 globally. Indeed, the evolution of SARS-CoV-2 was caused by the acquisition of several mutations since the pandemic started, as reported in the GISAID database (https://www.gisaid.org, accessed on 1 October 2020), which collected more than one million SARS-CoV-2 sequences. It is important to point out that the selection of random mutations stands out as one of the main mechanisms of acquiring resistance and represents a relevant phenomenon in viruses that mutate at high frequencies. RNA viruses, for instance, have a mutation rate estimated at 10^−4^ per nucleotide per replication [1,2]. In particular, the mutations were classified as variants of concern (VOCs), which included Alpha (B.1.1.7), Beta (B.1.351), Gamma (P.1), and Delta (B.1.617.2); variants of interest (VOI), comprising Eta (B.1.525), Iota (B.1.126), Kappa (B.1.617.1), Lambda (C.37), Omicron (BA.1), Zeta (P.2 (484 K.V2), and Ihu (B.1.640.1); and variants of alert (VOA) (https://www.who.int/en/activities/tracking-SARS-CoV-2-variants, accessed on 1 October 2020) [3,4]. However, this classification is dynamic, based on the spread of different variants over time and their clinical significance. In fact, these variants were able to emerge at the same time in multiple locations that are independent of each other, and then they were no longer circulating after a variable period. Chronologically, we have witnessed the emergence of B.1.1.7 in the United Kingdom (UK) [5], then B.1.351 in South Africa [6], followed by P.1 in Brazil [7], and B.1.617 in India [8]. These new variants show multiple mutations on their spike (S) glycoprotein and spread rapidly across the globe, resulting in more virulence. In December 2020, the B.1.1.7 variant was identified in the southeastern United Kingdom (UK), hence the name “UK variant”, and it is marked by 23 genetic mutations, compared to the original genome sequence that was first detected in Wuhan, China. 

As previously reported, other VOCs have also been isolated in South Africa, India, and Brazil and have been investigated for their enhanced contagiousness and resistance to neutralization. B.1.351 or the Beta variant that emerged in South Africa, is characterized by 18 mutations and 3 amino acid deletions in the S glycoprotein [9]. P.1 variant, also known as Gamma, was detected in December 2020 and has spread in an accelerated way across Manaus, Brazil. Another variant, identified as B.1.617, has been isolated from Maharashtra, India and is subdivided into three sub-lineages, such as B.1.617.1, B.1.617.2, and B.1.617.3 [10,11]. In November 2021, the emergence of the new variant detected in Botswana and South Africa, termed Omicron, alarmed the community due to the high number of changes in the spike protein, the increased transmission efficiency, and its ability to escape from neutralizing antibodies. However, clinical studies have reported that the rapidly spreading Omicron variant was less dangerous than its predecessor, the Delta variant [12]. The main differences found in this variant are to be attributed to the N-terminal domain (NTD): deletion of V143, Y144, and Y145 residues and a 3-amino-acid insertion of “EPE” at position 214. However, accumulating evidence suggests high transmission fitness due to its higher affinity to ACE2 receptors and efficient immune evasion, compared to the other VOCs [13].

To date, thanks to sequencing, the identification of emerging SARS-CoV-2 variants and sets of mutations potentially linked to changes in viral properties has been achieved. Due to these possible changes, the understanding of the functional consequences of VOCs and VOIs needs to rapidly expand. To do this, much of this knowledge should concern the impact of these variants in terms of conformational changes in the S glycoprotein, which could enhance receptor recognition and affect the production of neutralizing antibodies. 

Although assessing changes in the interaction between SARS-CoV-2 epitopes and antibodies is difficult, the binding affinity between mutated S glycoprotein and both T-cell receptor (TCR) and ACE2 receptor could be predicted in silico to test the immune response and infectivity, respectively. In this regard, computational methods hold the potential to understand resistance mechanisms, shedding light on the elusive link between novel mutations present in the S glycoprotein and its targets. These methods exploit the available structural information on protein–ligand complexes and structural modeling of point mutations in the protein structure [14]. Reported examples of the use of structure-based methods include: (i) the application of molecular docking to predict resistance or susceptibility of antiviral targets to different inhibitors [15,16]; (ii) the use of molecular dynamics simulations (MDs) to investigate the impact of mutations on enzyme stability and binding affinity on the receptor [17,18] or to highlight long-range altered communications in wild-type (WT) and mutated S glycoproteins; (iii) the use of computational mutation scanning protocols to extract insights on free energy and binding affinity changes, resulting from the active site and non-active site mutations [19]. Even though these methods are constantly adding new pieces to the puzzle and opening opportunities in the understanding of drug resistance, they suffer from various drawbacks, such as being time consuming and offering limited predictive accuracy. As a result of such limitations, the primary challenge facing structure-based drug resistance prediction is to achieve an acceptable balance between prediction accuracy and computational efficiency to become both reliable and fast tools to be used in the clinical context [17]. In fact, some of the most recent reports describe the use of machine learning strategies merging both sequence and structural data in an attempt to achieve such a balance [20,21]. In this perspective, several platforms have been developed to assemble sequencing data, provide information and tools to visualize the mutations present in the SARS-CoV-2 structures [22,23], and distribute a plethora of experimental data and bioinformatic tools through the European COVID-19 Data Platform (https://www.covid19dataportal.org/, accessed on 1 October 2020) for different purposes. Another SARS-CoV-2 immuno-analytics platform has been developed to visualize multidimensional data to inform target selection in immunological research by combining genomic and whole-proteome analyses with in silico epitope predictions [24,25]. Recently, in the context of drug discovery, user-friendly platforms were designed to identify promising drugs and targets for COVID-19, such as COVID19db [26] and COVID Moonshot [27]. Moreover, the EU-funded project, Exscalate4CoV (https://www.exscalate4cov.eu/, accessed on 1 October 2020), released additional web services, such as https://viralseq.exscalate4cov.eu/ and https://mediate.exscalate4cov.eu/ (accessed on 1 October 2020), to support the scientific community. The majority of created platforms collect data present in the literature and integrate databases that consider the SARS-CoV-2 targets, their mutations, and related clinical and immunological information. Unlike known platforms, we created an open access portal that provides original structural insights related to the SARS-CoV-2 spike variants. Our platform offers these mutated structures already modeled and previously equilibrated through molecular dynamic simulations, saving time and effort in the SARS-CoV-2 field of research. Therefore, we developed the Spike Mutants website (https://spikemutants.exscalate4cov.eu/, accessed on 1 October 2020), which contains the tridimensional coordinates of the mutated structures of the known VOCs and VOIs and their MDs trajectories. With the support of Exscalate4CoV, this platform acts as a solid starting point for more advanced studies, taking into account the impact of the mutations on the drug discovery process and the identification of effective strategies to plan new drugs and vaccines. Herein, we describe the main features of the portal and illustrate the possible applications for the scientific community.

## 2. Results

Unfortunately, with the rapid speed at which mutations occur in the S-protein, the crystal experimental structure of new variants is not feasible in a short period of time. In this context, computational models of the new variants can be developed for structural stability studies, design of new drugs, and baseline studies for the development of new vaccines, among others.

However, developing a realistic model of the new variants is a process that demands not only human time, but also computational time. 

For a better understanding of the complexity of building the models of the new variants, we detail some of the steps of our workflow: (1) analysis of the sequences of the new variants in order to determine which mutations are the most significant to be modeled; (2) homology modeling for the construction of a first 3D system based on the crystallographic structure of WT; (3) insertion of the glycans to the model in the correct position; (4) construction of the topology taking into account the correct description of the glycans force field and the new glycan–protein bonds that should be inserted into the new models; (5) MD system setup and simulations; (6) MD analysis. 

The complexity of this workflow is mainly due to the difficulty of inserting glycans covalently attached to the S protein and also to the equilibrium of the mutated systems, especially in the regions of insertion and deletion where secondary structures can be formed or undone.

For in silico experiments, an important aspect, which must not be neglected in the building of the 3D molecular model of the S glycoprotein of SARS-CoV-2, is the presence of numerous glycans on the surface of the protein. More specifically, the S glycoprotein of SARS-CoV-2 shows 18 N-glycosylation sequons [28] per monomer, two O-glycosylation [29] sites in the “head” (comprising S1/S2 subunits, residues 27–1140), and another four N-glycosylation sequons on the “stalk” portion of the protein (residues 1141–1234). Glycans not only determine the correct folding of the S glycoprotein but also shields the epitopes from antibody recognition [30], thus, modulating the structural and dynamical properties of protein [31]. In particular, the glycans on the “head” region of S glycoprotein are involved in the modulation of dynamics of the opening and closure of the protein [32,33,34] and in its capability to bind the ACE2 receptor. 

All these modeled structures were modeled and equilibrated through MDs and were collected in an open access portal to make these structures were available for the scientific community in order to represent a solid starting point for more advanced studies. 

**Figure 1:**
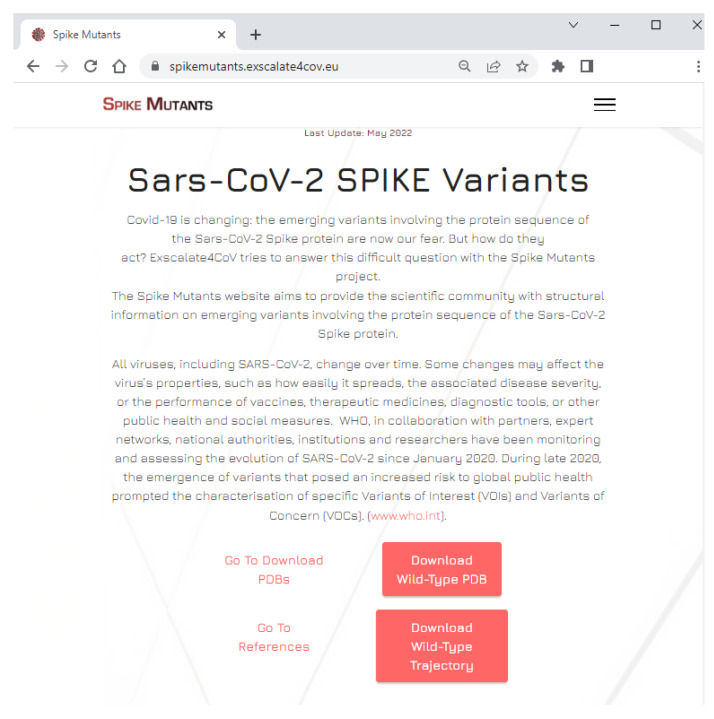
Snapshot of the upper portion of the Spike Mutants portal from where the 3D structure of the spike trimeric protein in glycosylated form and its corresponding MD trajectory 100 ns long can be freely downloaded.

The main page of the Spike Mutants portal (https://spikemutants.exscalate4cov.eu/, accessed on 1 October 2020) allows downloading the 3D structure of the spike trimeric protein in glycosylated form, as previously described [34], with its corresponding MD trajectory 100 ns long (see Figure 1) in PDB and GROMACS xtc format, respectively. 

**Figure 2:**
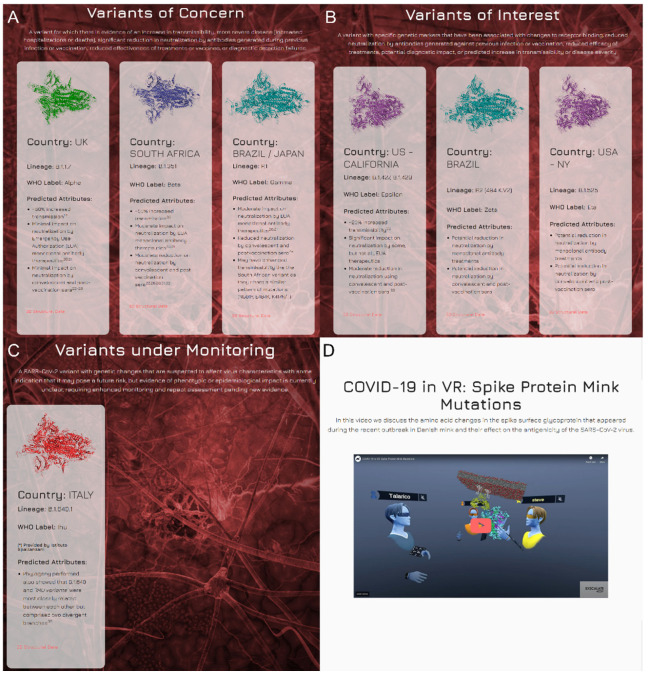
Snapshot of the Spike Mutants portal portions where the 3D structures of the spike protein unglycosylated variants can be freely downloaded. (**A**) “Variants of Concern” section; (**B**) “Variants of Interest” section; (**C**) “Variants under Monitoring” section; (**D**) virtual reality video of a 3D model of the S protein in interaction with the human receptor ACE2 embedded in a membrane bilayer.

The Spike Mutants website distributes 3D structures of SARS-CoV-2 S unglycosylated variants in three sections named “Variants of Concern”, “Variants of Interest”, and “Variants under Monitoring” (see Figure 2A–C, respectively) from which PDB structures can be freely downloaded.

**Figure 3:**
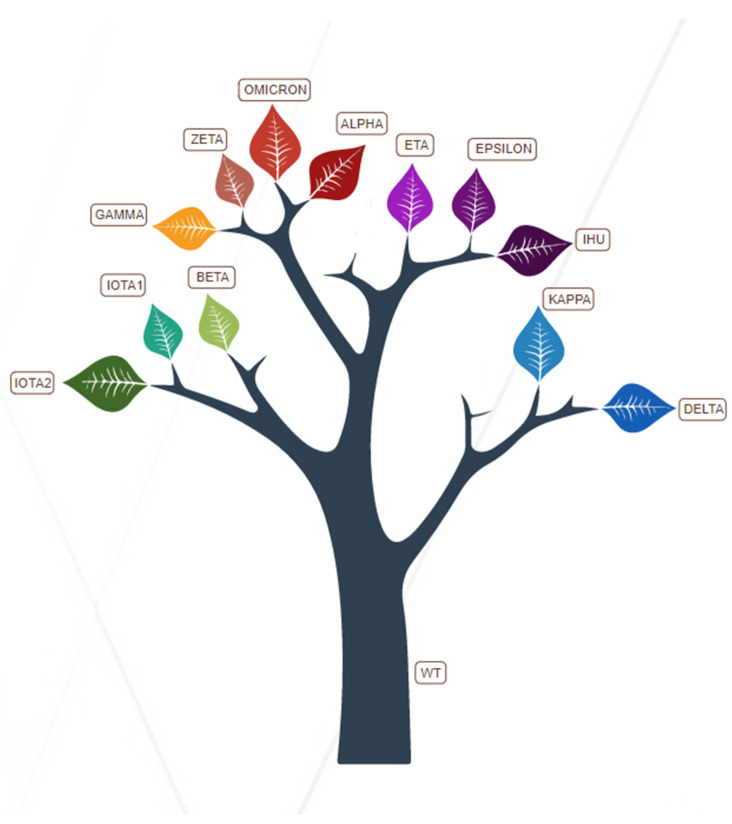
Graphical representation of the phylogenetic tree, included in the Spike Mutants portal constructed by the maximum likelihood method based on whole genome SARS-CoV-2 sequences.

On our website, it is also possible to visualize a graphical representation of the phylogenetic tree using the maximum likelihood method based on whole genome sequences of SARS-CoV-2 (Figure 3 and Figure S1) and a video of a 3D model of the S protein in interaction with the human receptor ACE2 embedded in a membrane bilayer (Figure 2D). 

**Figure 4:**
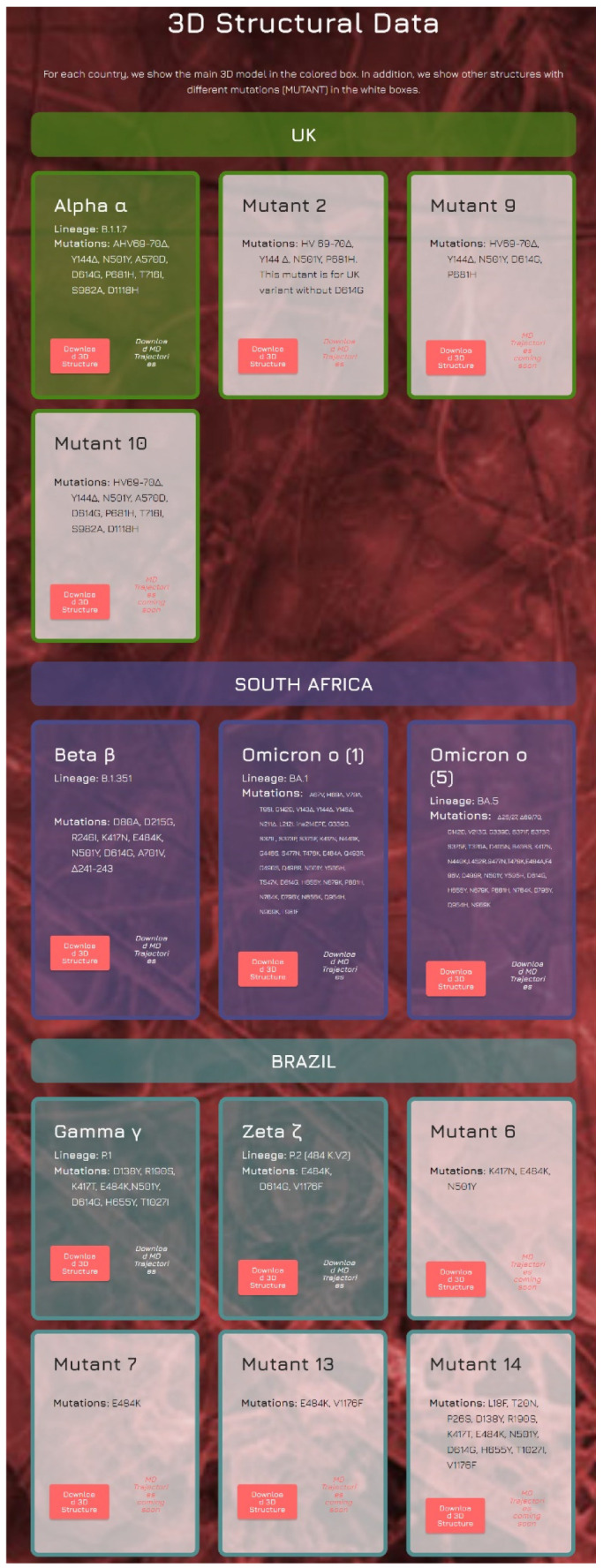
Snapshot of part of the Spike Mutants portal portions where the 3D structures of the spike protein glycosylated variants can be freely downloaded together with their respective MD trajectory, 100 ns long.

The last section of the Spike Mutants website distributes 3D models of the main SARS-CoV-2 S variants in glycosylated form, accompanied by their related MD trajectories (see Figure 4). All major WHO-labeled variants are present, highlighted in colored boxes. Their complete list is reported in Table 1 with “WHO Label”, lineage, and a list of mutations for each variant. In the same website section, additional sub-variants are distributed, highlighted with white boxes (Figure 4).

The modeling and simulation protocol is described in Materials and Methods. 

Together with the 3D model, through the web portal there is also a short MD trajectory available, 100 ns long, for each analyzed variant. Even though a better understanding of the structural and dynamic perturbation of the system is obtained with longer simulations (see results with one microsecond reported in recent works) [34,35,36], we found that interesting features can be inferred by them.

**Figure 5:**
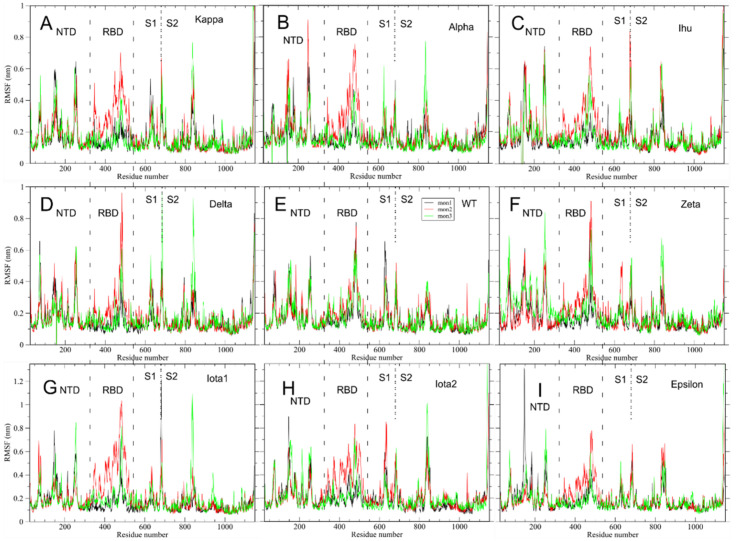
Per-residue RMSF in SARS-CoV-2 (**A**) Kappa, (**B**) Alpha, (**C**) Ihu, (**D**) Delta, (**E**) WT, (**F**) Zeta, (**G**) Iota1, (**H**) Iota2, (**I**) Epsilon systems. The three S monomers are colored in black, red, and green, respectively. Monomer 2 is in an up conformation (see Section 4). The NTD and RBD regions are highlighted with dashed lines. The S1/S2 boundary at residue 681 is highlighted with a dotted line.

The root main square fluctuations (RMSF) plot for some of the mutants in comparison to WT shows several examples (Figure 5).

(i) The monomer in the UP position (monomer 2, red in Figure 1) has higher fluctuations in a larger portion of its RBD in all mutants, as compared to WT, but, in particular, in some systems: i.e., Kappa, Alpha, Iota1, Iota2, and Epsilon (panel A, B, G, H and I, respectively);
(ii) Some of the mutants have higher RMSF peaks (higher than 10 Å) than WT and other mutants: Iota1, and Epsilon >12 Å; Iota2 > 10 Å (note that the *y*-axis has a different scale in panel G–I);
(iii) All mutants have higher fluctuations in the 834–853 region, namely the fusion-peptide proximal region (FPPR), as compared to WT and nearly all in monomer 3 (green in Figure 1), with the exception of Ihu (where monomer 2 and monomer 3 have comparable RMSF) and Epsilon (where monomer 2 has higher RMSF);
(iv) Several mutants have a higher RMSF peak at residue 681, at the boundary of S1 and S2, as compared to WT: i.e., Kappa, Ihu, Delta, and Iota1.

**Figure 6:**
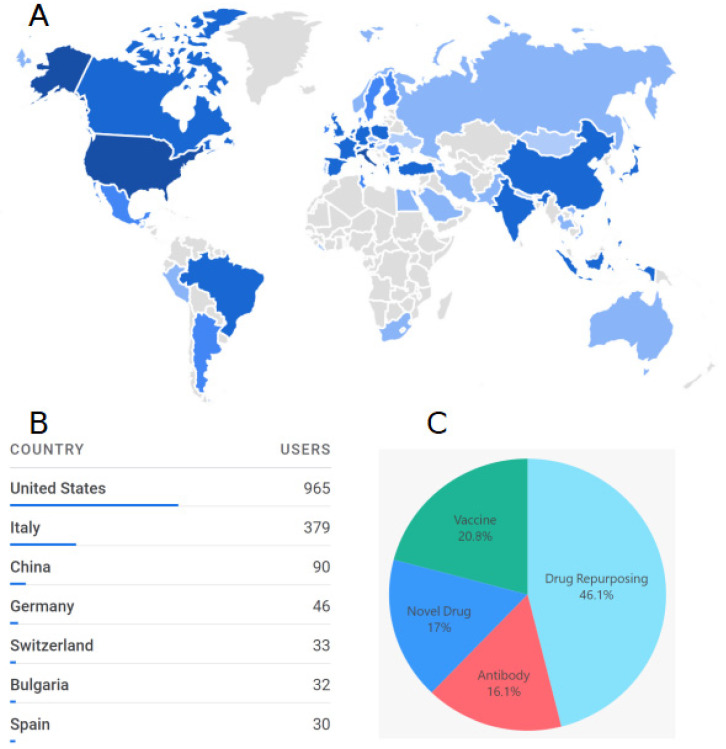
Registered users of the Spike Mutants website. (**A**) Country of origin of the users; (**B**) First seven countries by number of users. (**C**) Declared the main research objective of the users.

In the context of the EXSCALATE4COV (E4C) (www.exscalate4cov.eu, accessed on 1 October 2020) project, the Spike Mutants website was first made available in January 2021 as a public resource. From then, 1575 researchers from all over the world (see Figure 6A) registered on the website and downloaded the distributed structures and MD trajectories, with the USA being the country with the largest number of users (Figure 6B).

Regarding the main research objectives of the registered users, drug repurposing is the first subject, with 46% of users, while the vaccine is the second, with 20.9% of users (see Figure 6C). The reported numbers are constantly increasing. 

## 3. Discussion

With drug development being a series of crucial decisions to be considered to select the right target and binding site and to analyze the mutational pattern, it seems evident that computational chemistry can support each of those evaluations by capturing the value of simulation data into useful predictive models. In the context of COVID-19, the SARS-CoV-2 proteome has been deeply studied. One just has to think of all the tridimensional structures deposited in the Protein Data Bank, thus, discovering its complex mechanism and seeing the binding interactions with different chemical entities to accelerate the drug discovery process. Instead, regarding the virus’ genetic variability, the rate of onset of mutations on SARS-CoV-2 proteins makes it almost impossible to keep abreast. The emergence of new variants, as it is known, represents a major threat to public health. Indeed, they could compromise the therapeutic efficacy, developing resistance to antibodies and drugs [37]. Thus, the goal of this new platform is to provide a basis for further studies on how variants may influence the pandemic. Indeed, the scientific community could increase their knowledge in the fields of vaccines and antibodies by starting with the prepared models on our website and speeding up the drug repurposing and drug design of novel chemical entity processes. As evidence, this resource has recently been used to obtain effective results in the fields of neutralizing antibodies [38], studying the interaction between spike and aptamers [39], and providing important hints about spike protein mutant variants’ heparin interactions for potential resistance [40]. 

The Spike Mutants website provides a fast and easy way to understand the impact of the mutations on the binding affinity to human receptors and antibodies, aiming to enhance novel therapeutic and preventive solutions. Given the mutational profile’s fast pace of growth in the SARS-CoV-2 spike, we will promptly update the platform.

## 4. Materials and Methods

### 4.1. Data Storage and Web Implementation

The Spike Mutants website was developed to ensure sharing of our results. The website is based on the common LAMP software stack (Linux, Apache, MariaDB, PHP). The frontend interface was built on bootstrap 4, jQuery, and HTML5 doctype. The embedded survey service is SurveyJS.

### 4.2. Modeling of S Glycoproteins

The 3D protein models of the WT and the mutated spike were initially built by homology modeling using the web tool SWISS-MODEL [35]. The reference sequence used to align and model the missing atoms of the pre-fusion form of the cryo-EM structure (PDB ID: 6VYB) [36] was NCBI YP_009724390.1 (UniProt: P0DTC2 SPIKE_SARS2). In this structure, the RBD of the second monomer was in the up conformation, also known as open conformation, which favors the interaction with the ACE2 receptor. The full protocol of the WT model generation was described by Tagliamonte et al. [29]. For the variants, the mutated sequences taken into account were in accordance with the WHO classification and were reported in Table 1.

### 4.3. Mutant Modeling

The glycosylation of N- and O- sites of the WT and the variants (residues 27–1146) was built by using the glycoprotein builder available at GLYCAM-Web (www.glycam.org), as described by Borocci et al. [34]. Asymmetric glycosylation of the asparagine residue of the three monomers of the WT and the variant protein was used, as reported in Tables S1–S3. The presence of steric clashes between atoms of protein and glycans was removed by changing the dihedrals of the N–glycosidic bond.

### 4.4. Glycosylation of S Proteins

The modeled spike glycoprotein structure of the WT and the variants (residues 27–1146) contains N-glycosylation [28] and O-glycans [29] sites and was constructed, as described by Borocci et al. [34]. Tables S1–S3 detail the glycans that are bounded with the spike amino acids. 

### 4.5. Molecular Dynamics Simulation and Analyses

The full MD protocol is described in previous work [34]. Briefly, the forcefields Amber14SB [41], GLYCAM06 [42], and TIP3P [43] were employed to model the protein, the glycans, and the water molecules, respectively. The ACPYPE script [44] was used to convert the topology into the GROMACS format. All the MD simulations were run with GROMACS 2020.6 package [45]. The P-LINCS [46,47] algorithm was used to restrain h-bonds and the SETTLE [48] algorithm was employed to constrain the water molecules. The particle mesh Ewald method (PME) [49] was used to model the short-range interactions. The systems were firstly minimized in vacuum, applying harmonic potential restraints of 1000 kJ mol^−1^ nm^−2^ to the protein atoms. Then, cubic simulation boxes were created and neutralized. Unrestrained MD simulations were performed with a time step of 2 fs and with a length of 100 ns for each system. The 300 K temperature and the 1 bar pressure were controlled, respectively, by V-rescale [50] and Parrinello–Rahman barostat algorithms [51]. For the RMSF analyses, which were performed by the GROMACS RMSF tool, the first 5 ns of each trajectory were excluded. For the variants with deletion, dummy residues with null fluctuations were inserted in the RMSF plot to preserve the WT residue numbering. All the simulations and analyses were performed on the supercomputer Marconi-100, CINECA, Bologna, Italy.

### 4.6. Phylogenetic Analyses

SARS-CoV-2 genome sequences were retrieved from GISAID [52] and high-quality, full-length genome representatives for main lineages were analyzed as follows. Whole-genome alignment was performed with MAFFT v7.271 [53]; multi-sequence alignment was then manually controlled and 5′ and 3′ UTR regions were excluded from further analysis. Maximum likelihood (ML) phylogenetic analysis was performed with IQ-TREE v.1.6.12 [54]; the best tree model was selected using ModelFinder [55]; the best trees were found performing 5000 bootstrap ultrafast replicates. Strain “Whuan-Hu-1” was used as a phylogenetic outgroup (PANGO-lineage B, NCBI Acc. Numb. NC_045512.2).

## Figures and Tables

**Table 1:** SARS-CoV-2 spike variants whose 3D models and MD trajectories are distributed through the web portal.

WHO Label	Lineage	List of Mutations
Alpha	B.1.1.7	HV69-70Δ, Y144Δ, N501Y, A570D, D614G, P681H, T716I, S982A, D1118H
Beta	B.1.351	D80A, D215G, R246I, K417N, E484K, N501Y, D614G, A701V, LAL242-244Δ
Delta	B.1.617.2	T19R, EE156-157Δ, R158G, L452R, T478K, D614G, P681R, D950N
Epsilon	B.1.427, B.1.429	S13I, W152C, L452R, D614G
Eta	B.1.525	Q52R, A67V, HV69-70Δ, Y144Δ, E484K, D614G, Q677H, F888L
Gamma	P.1	L18F, T20N, P26S, D138Y, R190S, K417T, E484K, N501Y, D614G, H655Y, T1027I, V1176F
Iota1	B.1.526	T95I, D253G, S477N, D614G, A701V
Iota2	B.1.526	T95I, D253G, E484K, D614G, A701V
Kappa	B.1.617.1, B.1.617.3	E154K, E484Q, D614G, P681R, Q1071H
Omicron	BA.1	A67V, HV69-70Δ, T95I, G142D, VYY143-145Δ, N211Δ, L212I, EPE214ins, G339D, S371L, S373P, S375F, K417N, N440K, G446S, S477N, T478K, E484A, Q493R, G496S, Q498R, N501Y, Y505H, T547K, D614G, H655Y, N679K, P681H, N764K, D796Y, N856K, Q954H, N969K, L981F
	BA.5	PPA25-27Δ, HV69-70Δ, G142D, V213G, G339D, S371F, S373P, S375F, T376A, D405N, R408S, K417N, N440K, L452R, S477N, T478K, E484A, F486V, Q498R, N501Y, Y505H, D614G, H655Y, N679K, P681H, N764K, D796Y, Q954H, N969K
Zeta	P.2 (484 K.V2)	E484K, D614G, V1176F
Ihu *	B.1.640.1	P9L; E96Q; CNDPFLGVY136-144Δ; R190S; I210T; R346S; N394S; Y449N; F490R; N501Y; D614G; P681H; T859N; D936H; K1191N

Note that the first residue in the models is number 27 and the higher is 1146, not considering the glycosyl groups. Therefore, the variants outside this range are reported only for the sake of completeness. * Not a WHO Label.

## Data Availability

Not applicable.

## Supplementary Materials

The supporting information can be downloaded at: https://www.mdpi.com/article/10.3390/ijms232113082/s1.

## References

[1] Tibayrenc M. (2017). Genetics and Evolution of Infectious Diseases.

[2] Hodge A.V., Field H.J. (2011). General mechanisms of antiviral resistance. Genetics and Evolution of Infectious Disease.

[3] Fontanet A., Autran B., Lina B., Kieny M.P., Karim S.S.A., Sridhar D. (2021). SARS-CoV-2 variants and ending the COVID-19 pandemic. Lancet.

[4] Grubaugh N.D., Hanage W.P., Rasmussen A.L. (2020). Making sense of mutation: What D614G means for the COVID-19 pandemic remains unclear. Cell.

[5] Tang J.W., Tambyah P.A., Hui D.S. (2021). Emergence of a new SARS-CoV-2 variant in the UK. J. Infect..

[6] Tegally H., Wilkinson E., Giovanetti M., Iranzadeh A., Fonseca V., Giandhari J., Doolabh D., Pillay S., San E.J., Msomi N. (2020). Emergence and rapid spread of a new severe acute respiratory syndrome-related coronavirus 2 (SARS-CoV-2) lineage with multiple spike mutations in South Africa. Nature.

[7] Naveca F., da Costa C., Nascimento V., Souza V., Corado A., Nascimento F., Costa Á., Duarte D., Silva G., Mejía M. (2021). SARS-CoV-2 reinfection by the new Variant of Concern (VOC) P. 1 in Amazonas, Brazil. https://virological.org/.

[8] Samarasekera U. (2021). India grapples with second wave of COVID-19. Lancet Microbe.

[9] Boehm E., Kronig I., Neher R.A., Eckerle I., Vetter P., Kaiser L. (2021). Novel SARS-CoV-2 variants: The pandemics within the pandemic. Clin. Microbiol. Infect..

[10] Srivastava S., Banu S., Singh P., Sowpati D.T., Mishra R.K. (2021). SARS-CoV-2 genomics: An Indian perspective on sequencing viral variants. J. Biosci..

[11] Jung C., Kmiec D., Koepke L., Zech F., Jacob T., Sparrer K.M., Kirchhoff F. (2022). Omicron: What makes the latest SARS-CoV-2 variant of concern so concerning?. J. Virol..

[12] Maslo C., Friedland R., Toubkin M., Laubscher A., Akaloo T., Kama B. (2022). Characteristics and outcomes of hospitalized patients in South Africa during the COVID-19 Omicron wave compared with previous waves. JAMA.

[13] Hao G.-F., Yang G.-F., Zhan C.-G. (2012). Structure-based methods for predicting target mutation-induced drug resistance and rational drug design to overcome the problem. Drug Discov. Today.

[14] Pouga L., Santoro M.M., Charpentier C., Di Carlo D., Romeo I., Artese A., Alcaro S., Antinori A., Wirden M., Perno C.F. (2019). New resistance mutations to nucleoside reverse transcriptase inhibitors at codon 184 of HIV-1 reverse transcriptase (M184L and M184T). Chem. Biol. Drug Des..

[15] Romeo I., Marascio N., Pavia G., Talarico C., Costa G., Alcaro S., Artese A., Torti C., Liberto M.C., Focà A. (2018). Structural Modeling of New Polymorphism Clusters of HCV Polymerase Isolated from Direct-Acting Antiviral Naïve Patients: Focus on Dasabuvir and Setrobuvir Binding Affinity. ChemistrySelect.

[16] Lupia A., Moraca F., Bagetta D., Maruca A., Ambrosio F.A., Rocca R., Catalano R., Romeo I., Talarico C., Ortuso F. (2020). Computer-based techniques for lead identification and optimization II: Advanced search methods. Phys. Sci. Rev..

[17] Marascio N., Pavia G., Romeo I., Talarico C., Di Salvo S., Reale M., Marano V., Barreca G.S., Fabiani F., Perrotti N. (2018). Real-life 3D therapy failure: Analysis of NS5A 93H RAS plus 108 K polymorphism in complex with ombitasvir by molecular modeling. J. Med. Virol..

[18] Hao G.-F., Yang G.-F., Zhan C.-G. (2010). Computational mutation scanning and drug resistance mechanisms of HIV-1 protease inhibitors. J. Phys. Chem. B.

[19] Masso M., Vaisman I.I. (2013). Sequence and structure based models of HIV-1 protease and reverse transcriptase drug resistance. BMC Genom..

[20] Alves N.G., Mata A.I., Luís J.P., Brito R.M., Simões C.J. (2020). An Innovative Sequence-to-Structure-Based Approach to Drug Resistance Interpretation and Prediction: The Use of Molecular Interaction Fields to Detect HIV-1 Protease Binding-Site Dissimilarities. Front. Chem..

[21] Sedova M., Jaroszewski L., Alisoltani A., Godzik A. (2020). Coronavirus3D: 3D structural visualization of COVID-19 genomic divergence. Bioinformatics.

[22] Phelan J., Deelder W., Ward D., Campino S., Hibberd M.L., Clark T.G. (2022). COVID-profiler: A webserver for the analysis of SARS-CoV-2 sequencing data. BMC Bioinform..

[23] Ward D., Higgins M., Phelan J.E., Hibberd M.L., Campino S., Clark T.G. (2021). An integrated in silico immuno-genetic analytical platform provides insights into COVID-19 serological and vaccine targets. Genome Med..

[24] Murdocca M., Citro G., Romeo I., Lupia A., Miersch S., Amadio B., Bonomo A., Rossi A., Sidhu S.S., Pandolfi P.P. (2021). Peptide Platform as a Powerful Tool in the Fight against COVID-19. Viruses.

[25] Feng Z., Chen M., Liang T., Shen M., Chen H., Xie X.-Q. (2021). Virus-CKB: An integrated bioinformatics platform and analysis resource for COVID-19 research. Brief. Bioinform..

[26] Zhang W., Zhang Y., Min Z., Mo J., Ju Z., Guan W., Zeng B., Liu Y., Chen J., Zhang Q. (2022). COVID19db: A comprehensive database platform to discover potential drugs and targets of COVID-19 at whole transcriptomic scale. Nucleic Acids Res..

[27] Chodera J., Lee A.A., London N., von Delft F. (2020). Crowdsourcing drug discovery for pandemics. Nat. Chem..

[28] Watanabe Y., Allen J.D., Wrapp D., McLellan J.S., Crispin M. (2020). Site-specific glycan analysis of the SARS-CoV-2 spike. Science.

[29] Shajahan A., Supekar N.T., Gleinich A.S., Azadi P. (2020). Deducing the N-and O-glycosylation profile of the spike protein of novel coronavirus SARS-CoV-2. Glycobiology.

[30] Grant O.C., Montgomery D., Ito K., Woods R.J. (2020). Analysis of the SARS-CoV-2 spike protein glycan shield reveals implications for immune recognition. Sci. Rep..

[31] Chawla H., Fadda E., Crispin M. (2022). Principles of SARS-CoV-2 Glycosylation. Curr. Opin. Struct. Biol..

[32] Harbison A.M., Fogarty C.A., Phung T.K., Satheesan A., Schulz B.L., Fadda E. (2022). Fine-tuning the spike: Role of the nature and topology of the glycan shield in the structure and dynamics of the SARS-CoV-2 S. Chem. Sci..

[33] Casalino L., Gaieb Z., Goldsmith J.A., Hjorth C.K., Dommer A.C., Harbison A.M., Fogarty C.A., Barros E.P., Taylor B.C., McLellan J.S. (2020). Beyond shielding: The roles of glycans in the SARS-CoV-2 spike protein. ACS Cent. Sci..

[34] Borocci S., Cerchia C., Grottesi A., Sanna N., Prandi I.G., Abid N., Beccari A.R., Chillemi G., Talarico C. (2021). Altered Local Interactions and Long-Range Communications in UK Variant (B.1.1.7) Spike Glycoprotein. Int. J. Mol. Sci..

[35] Tagliamonte M., Abid N., Borocci S., Sangiovanni E., Ostrov D., Kosakovsky Pond S.L., Salemi M., Chillemi G., Mavian C. (2021). Multiple recombination events and strong purifying selection at the origin of SARS-CoV-2 spike glycoprotein increased correlated dynamic movements. Int. J. Mol. Sci.

[36] Prandi I.G., Mavian C., Giombini E., Gruber C.E., Pietrucci D., Borocci S., Abid N., Beccari A.R., Talarico C., Chillemi G. (2022). Structural Evolution of Delta (B.1.617.2) and Omicron (BA. 1) Spike Glycoproteins. Int. J. Mol. Sci..

[37] Burki T. (2021). Understanding variants of SARS-CoV-2. Lancet.

[38] Gattinger P., Niespodziana K., Stiasny K., Sahanic S., Tulaeva I., Borochova K., Dorofeeva Y., Schlederer T., Sonnweber T., Hofer G. (2022). Neutralization of SARS-CoV-2 requires antibodies against conformational receptor-binding domain epitopes. Allergy.

[39] Villa A., Brunialti E., Dellavedova J., Meda C., Rebecchi M., Conti M., Donnici L., De Francesco R., Reggiani A., Lionetti V. (2022). DNA aptamers masking angiotensin converting enzyme 2 as an innovative way to treat SARS-CoV-2 pandemic. Pharmacol. Res..

[40] Gupta Y., Maciorowski D., Zak S.E., Kulkarni C.V., Herbert A.S., Durvasula R., Fareed J., Dye J.M., Kempaiah P. (2021). Heparin: A simplistic repurposing to prevent SARS-CoV-2 transmission in light of its in-vitro nanomolar efficacy. Int. J. Biol. Macromol..

[41] Maier J.A., Martinez C., Kasavajhala K., Wickstrom L., Hauser K.E., Simmerling C. (2015). ff14SB: Improving the accuracy of protein side chain and backbone parameters from ff99SB. J. Chem. Theory Comput..

[42] Kirschner K.N., Yongye A.B., Tschampel S.M., González-Outeiriño J., Daniels C.R., Foley B.L., Woods R.J. (2008). GLYCAM06: A generalizable biomolecular force field. Carbohydrates. J. Comput. Chem..

[43] Mark P., Nilsson L. (2001). Structure and dynamics of the TIP3P, SPC, and SPC/E water models at 298 K. J. Phys. Chem. A.

[44] Sousa da Silva A.W., Vranken W.F. (2012). ACPYPE-Antechamber python parser interface. BMC Res. Notes.

[45] Páll S., Zhmurov A., Bauer P., Abraham M., Lundborg M., Gray A., Hess B., Lindahl E. (2020). Heterogeneous parallelization and acceleration of molecular dynamics simulations in GROMACS. J. Chem. Phys..

[46] Hess B. (2008). P-LINCS: A parallel linear constraint solver for molecular simulation. J. Chem. Theory Comput..

[47] Hess B., Bekker H., Berendsen H.J., Fraaije J.G. (1997). LINCS: A linear constraint solver for molecular simulations. J. Comput. Chem..

[48] Miyamoto S., Kollman P.A. (1992). Settle: An analytical version of the SHAKE and RATTLE algorithm for rigid water models. J. Comput. Chem..

[49] Darden T., York D., Pedersen L. (1993). Particle mesh Ewald: An *N*·log(*N*) method for Ewald sums in large systems. J. Chem. Phys..

[50] Bussi G., Donadio D., Parrinello M. (2007). Canonical sampling through velocity rescaling. J. Chem. Phys..

[51] Parrinello M., Rahman A. (1981). Polymorphic transitions in single crystals: A new molecular dynamics method. J. Appl. Phys..

[52] Khare S., Gurry C., Freitas L., Schultz M., Bach G., Diallo A., Akite N., Ho J., Lee R., Yeo W. (2021). GISAID’s Role Pandemic Response. China CDC Wkly.

[53] Katoh K., Toh H. (2010). Parallelization of the MAFFT multiple sequence alignment program. Bioinformatics.

[54] Trifinopoulos J., Nguyen L.-T., von Haeseler A., Minh B.Q. (2016). W-IQ-TREE: A fast online phylogenetic tool for maximum likelihood analysis. Nucleic Acids Res..

[55] Kalyaanamoorthy S., Minh B.Q., Wong T.K., Von Haeseler A., Jermiin L.S. (2017). ModelFinder: Fast model selection for accurate phylogenetic estimates. Nat. Methods.

