# Supplementary material for: The Spike Mutants Website: A Worldwide Used Resource against SARS-CoV-2
Source: Int J Mol Sci. 2022 Oct 28;23(21):13082. doi: 10.3390/ijms232113082 (PMC9654198; doi:10.3390/ijms232113082)
Supplement: Supplementary file 1 [file ijms-23-13082-s001.zip › ijms-1956394-supplementary.pdf]

Supplementary Materials

The Spike Mutants website: a worldwide used resource against SARS-CoV-2

Isabella Romeo[1,2]†, Ingrid Guarnetti Prandi[3]†, Emanuela Giombini[4], Cesare Ernesto Maria Gruber[4], Daniele Pietrucci[3,5], Stefano Borocci[3,6], Nabil Abid[6,7], Anna Fava[8], Andrea R. Beccari[8], Giovanni Chillemi[3], Carmine Talarico[8,*]

[1]Dipartimento di Scienze della Salute, Università Magna Græcia di Catanzaro, Viale Europa Campus "S. Venuta", Viale Europa, 88100 Catanzaro, Italy, isabella.romeo@unicz.it (I. R.)

[2]Net4Science Academic Spin-Off, Università Magna Graecia di Catanzaro, Campus "S. Venuta", Viale Europa, 88100 Catanzaro, Italy. isabella.romeo@unicz.it (I. R.)

[3]Department for Innovation in Biological, Agro-food and Forest systems, DIBAF, University of Tuscia, via S. Camillo de Lellis s.n.c., 01100 Viterbo, Italy; ingrid.prandi@unitus.it (I.G.P.); borocci@unitus.it (S.B.); gchillemi@unitus.it (G.C.)

[4]Laboratory of Virology, INMI Lazzaro Spallanzani IRCCS, via Portuense 292, 00149, Roma, Italy; emanuela.giombini@inmi.it (E.G.), cesare.gruber@inmi.it (C.G.)

[5]Institute of Biomembranes, Bioenergetics and Molecular Biotechnologies, IBIOM, CNR, 70126 Bari, Italy, d.pietrucci@ibiom.cnr.it (D.P.)

[6]Laboratory of Transmissible Diseases and Biological Active Substances LR99ES27, Faculty of Pharmacy, University of Monastir, Rue Ibn Sina, 5000, Monastir, Tunisia; nabilabidbensalem.2014@yahoo.fr (N.A)

[7]High Institute of Biotechnology of Monastir, Department of Molecular and Cellular Biology, University of Monastir, Monastir, Tunisia; nabilabidbensalem.2014@yahoo.fr (N.A)

[8]Dompé Farmaceutici SpA, Via Tommaso De Amicis, 95, Napoli, 80131, Italy; carmine.talarico@dompe.com (C.T.); andrea.beccari@dompe.com (A.R.B.); anna.fava@dompe.com (A.F.)

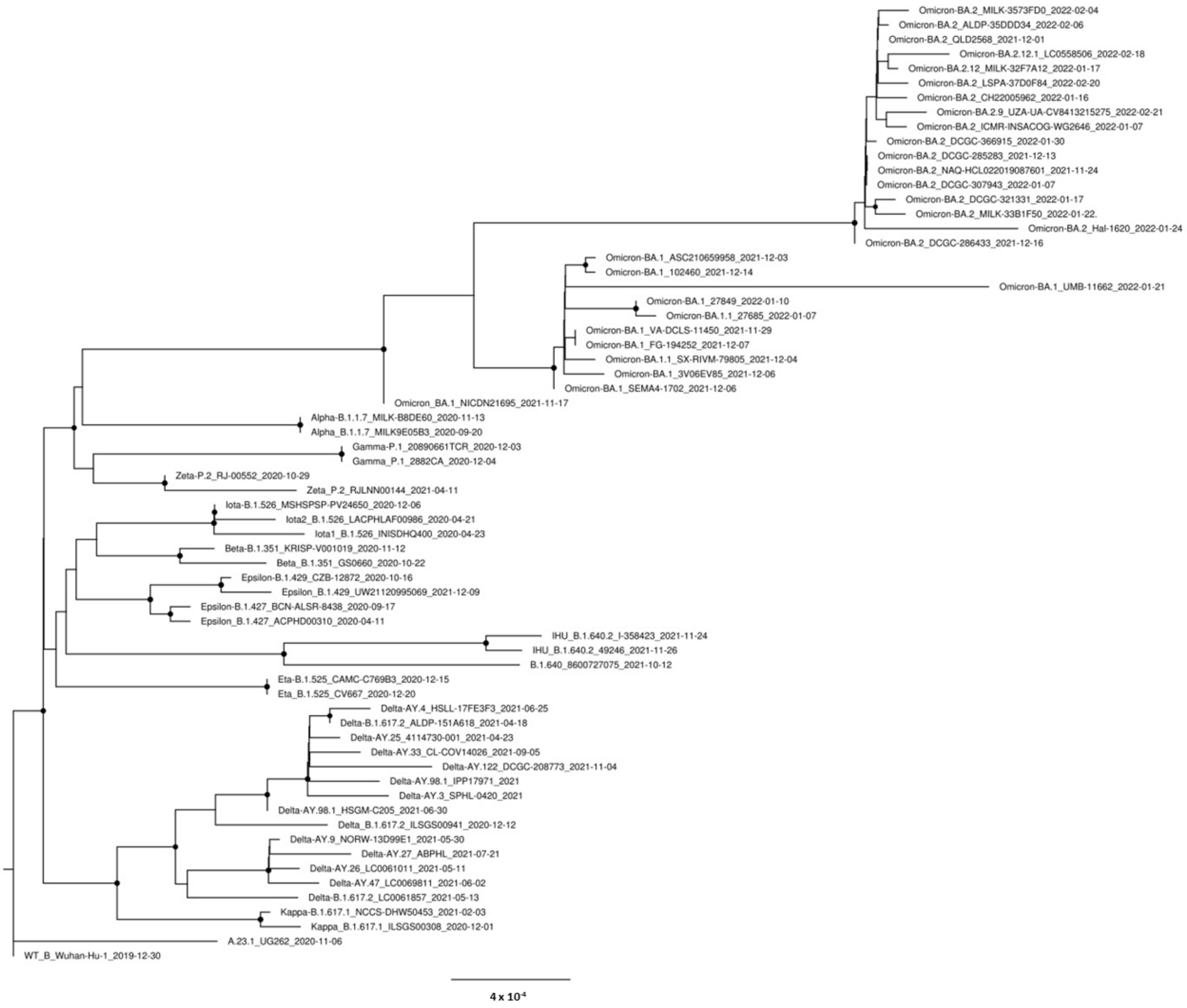

Figure S1: Phylogenetic tree constructed by the Maximum likelihood method based on the whole genome sequences. Nodes supported with bootstrap values ≥80 are marked with black dots.

Table S1. Glycan composition of monomer 1 of SARS-CoV-2 S glycoprotein

Residue	ID	Type	Structure
N61	glyc_1	M5	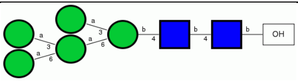
N74	glyc_2	A3	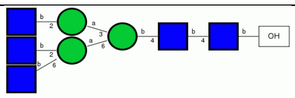
N122	glyc_3	M5	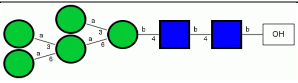
N149	glyc_4	FA2G2S1	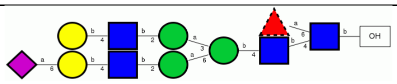
N165	glyc_5	FA2G2S2	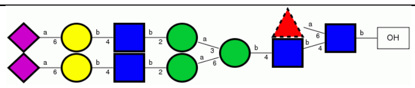
N234	glyc_6	M8	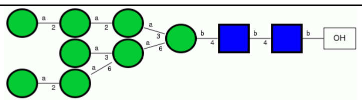
N282	glyc_7	FA3	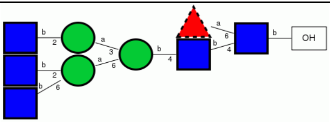
N331	glyc_8	FA2	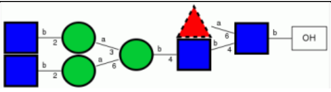
N343	glyc_9	FA2	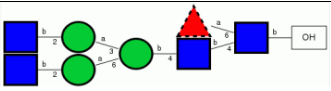
N603	glyc_10	FA2	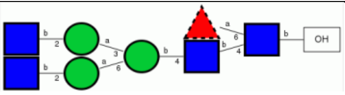
N616	glyc_11	A2	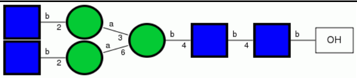
N657	glyc_12	M5	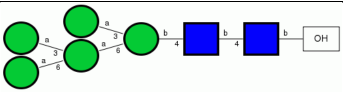
N709	glyc_13	M6	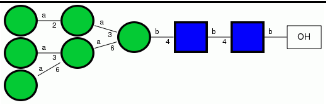
N717	glyc_14	Hybrid G1	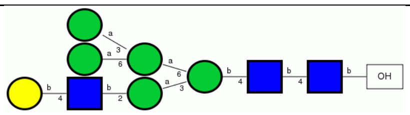

N801	glyc_15	M6	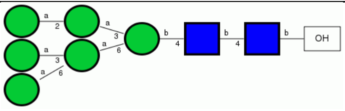
N1074	glyc_16	FA2G2S1	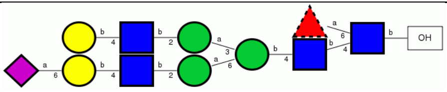
N1098	glyc_17	FA2	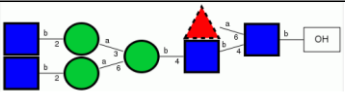
N1134	glyc_18	FA1	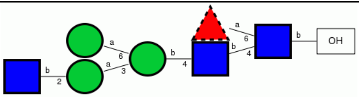
T323	glyc_19	O-glycan[a]	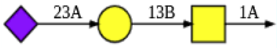
S325	glyc_20	O-glycan[a]	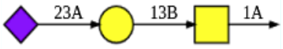

Table S2. Glycan composition of monomer 2 ("up" conformation) of SARS-CoV-2 S glycoprotein

Residue	ID	Type	Structure
N61	glyc_1	M5	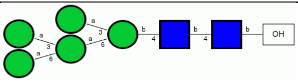
N74	glyc_2	FA3G3S2	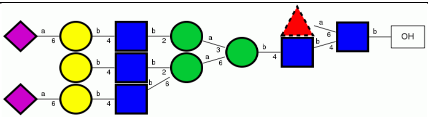
N122	glyc_3	FA2	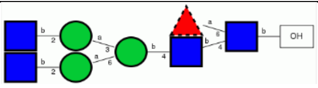
N149	glyc_4	FA3	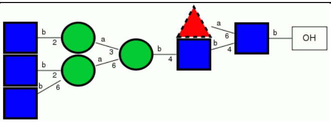
N165	glyc_5	M5	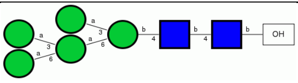
N234	glyc_6	M9	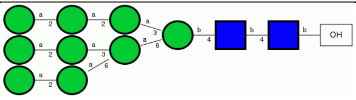
N282	glyc_7	FA3G3S1	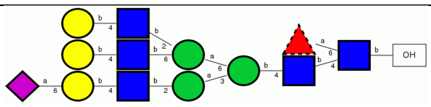

N331	glyc_8	FA2	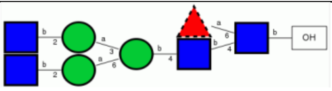
N343	glyc_9	FA1	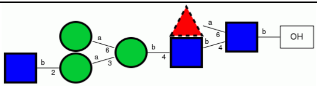
N603	glyc_10	M5	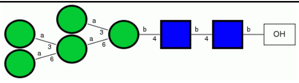
N616	glyc_11	FA2	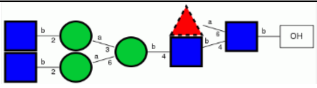
N657	glyc_12	Hybrid G1	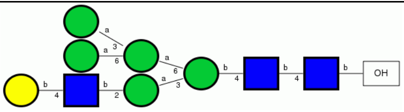
N709	glyc_13	M5	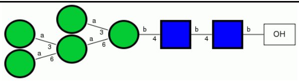
N717	glyc_14	M5	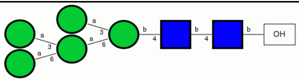
N801	glyc_15	M7	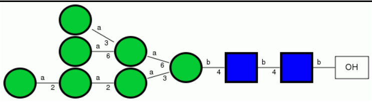
N1074	glyc_16	M5	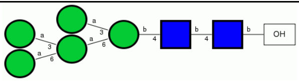
N1098	glyc_17	A2	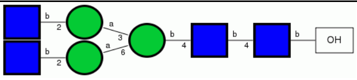
N1134	glyc_18	FA3	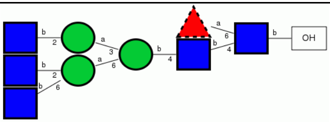
T323	glyc_19	O-glycan[b]	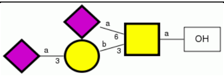

Table S3. Glycan composition of monomer 3 of SARS-CoV-2 S glycoprotein

**Residue**	**ID**	**Type**	**Structure**
N61	glyc_1	M5	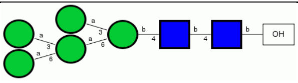

N74	glyc_2	A2	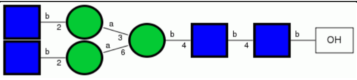
N122	glyc_3	M5	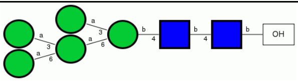
N149	glyc_4	FA2	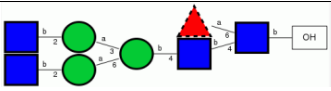
N165	glyc_5	FA2G2S1	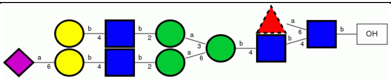
N234	glyc_6	M9	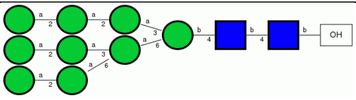
N282	glyc_7	A2	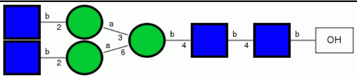
N331	glyc_8	FA3G3S1	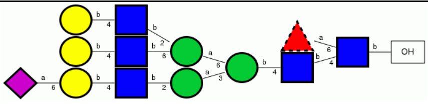
N343	glyc_9	FA2	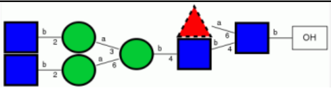
N603	glyc_10	M5	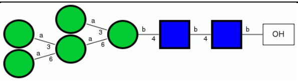
N616	glyc_11	FA2	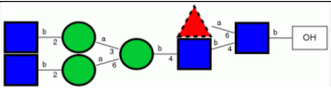
N657	glyc_12	Hybrid G1	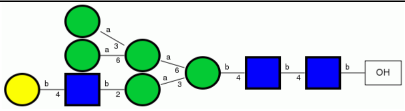
N709	glyc_13	M5	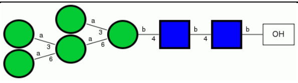
N717	glyc_14	M6	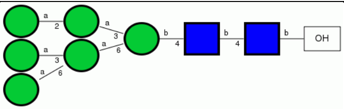
N801	glyc_15	M5	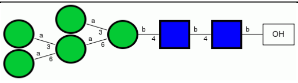
N1074	glyc_16	M5	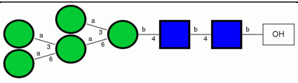

N1098	glyc_17	Hybrid G1S1	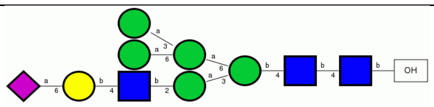
N1134	glyc_18	FA2	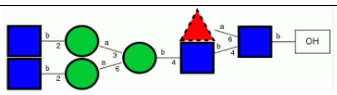
T323	glyc_19	O-glycan[c]	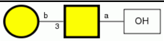

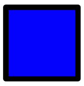 N-acetylglucosamine

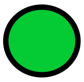 Mannose

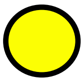 Galactose

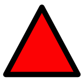 Fucose

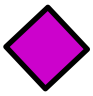 N-acetylneuraminic acid
